# Glutaminase inhibition is correlated with an increase in phospholipid unsaturation, a potential cellular adaptation to pH fluctuations

**DOI:** 10.1038/s41598-026-45555-5

**Published:** 2026-04-03

**Authors:** Soichiro Miyamoto, Kana Matsumoto, Hiroyuki Saito, Kohjiro Nagao

**Affiliations:** 1 Department of Synthetic Chemistry and Biological Chemistry, Graduate School of Engineering, Kyoto University, Kyoto, 615-8510 Japan; 2 Laboratory of Biophysical Chemistry, Kyoto Pharmaceutical University, Misasaginakauchi-cho, Yamashina-ku, Kyoto, 607-8414 Japan

**Keywords:** Glutaminase, Phospholipid, pH, Monounsaturated fatty acid, Δ9-fatty acid desaturase, Biochemistry, Cell biology, Physiology

## Abstract

The ability of cells to adapt to stress is fundamental for the maintenance of cellular homeostasis. In this study, we identified a possible adaptation mechanism to pH fluctuations while investigating the role of glutamine metabolism in *Drosophila* S2 cells. Inhibition of glutaminase (GLS), an enzyme that catalyzes the deamination of glutamine to glutamate, yielding ammonia, has been correlated with an increase in monounsaturated fatty acid (MUFA) content in membrane phospholipids. GLS inhibition–linked lipid remodeling was driven by the selective promotion of MUFA-rich phospholipid biosynthesis and was reversed by the addition of basic compounds such as ammonia, a byproduct of the GLS reaction, or NaOH, but not by glutamate-derived metabolites. Furthermore, lowering the pH of culture medium with HCl or reducing intracellular pH through the inhibition of the Na⁺/H⁺ exchanger resulted in lipid composition changes similar to those observed in GLS-inhibited cells. This suggests that pH changes govern membrane lipid unsaturation and that cellular acidification itself promotes the accumulation of MUFA-rich phospholipids. Notably, cells with higher MUFA levels exhibited higher intracellular pH than those with lower MUFA levels. Taken together, these findings indicate that cells respond to pH fluctuations by adjusting membrane lipid unsaturation to maintain cellular pH homeostasis.

**Supplementary Information:**

The online version contains supplementary material available at 10.1038/s41598-026-45555-5.

## Introduction

Phospholipids play a pivotal role in establishing and maintaining the cell membrane architecture. Glycerophospholipids, which are the predominant phospholipids in cell membranes, consist of a glycerol backbone linked to a phosphate-containing polar head group and two strands of fatty acyl chains^1^. The structural diversity of both the polar head group and fatty acyl chains enables cells to generate a wide array of phospholipid species^2^. Notably, cellular phospholipids exhibit remarkable variations in the combination of fatty acyl chains. These fatty acids can be categorized into three groups based on the number of double bonds in their acyl chains: saturated fatty acids (SFAs), which contain no double bonds; monounsaturated fatty acids (MUFAs), which contain one double bond; and polyunsaturated fatty acids (PUFAs), which contain two or more double bonds. The presence of double bonds introduces kinks in fatty acyl chains, influencing the molecular behavior of phospholipid molecules and their interactions with neighboring membrane constituents^3,4^. Thus, the degree of unsaturation of phospholipid acyl chains modulates the physicochemical properties of bilayer membranes and the functionality of membrane-associated proteins^5–7^.

The dynamic remodeling of cellular constituents in response to intrinsic and extrinsic stress is a key adaptive mechanism that supports cellular homeostasis. One key aspect of this adaptive process is the reorganization of phospholipid molecules^3^ because the specific fatty acid composition of cellular phospholipids confers adaptive advantages under fluctuating cellular conditions. In ectothermic animals, higher levels of unsaturated fatty acids help maintain the membrane environment at lower temperatures by enhancing the bilayer fluidity^8^. Because PUFAs in phospholipid acyl chains enhance membrane deformability^4,5^, increased PUFA content facilitates adaptation to osmotic stress by modulating the mechanical properties of the cell membrane^9^. Thus, maintaining an optimal phospholipid composition is essential for cellular adaptation to environmental stimuli and preservation of physiological cellular functions. However, the cellular mechanisms that regulate the phospholipid composition in response to fluctuating cellular conditions have not yet been fully elucidated.

In most mammals, distinct fatty acid desaturases introduce *cis* double bonds at the Δ5-, Δ6-, and Δ9-positions of the fatty acid moiety of acyl-CoA^10^. These desaturases enable the mammalian cells to produce various fatty acids in a situation-dependent manner^11–13^. In contrast, *Drosophila melanogaster* encodes only a Δ9-fatty acid desaturase, which introduces a *cis* double bond at the Δ9-position^14^. Consequently, *Drosophila* can only synthesize a limited set of fatty acids. In the commonly used *Drosophila* cell line S2, DESAT1 functions as the sole Δ9-fatty acid desaturase^7^. Moreover, S2 cells exhibited minimal uptake of PUFAs from the extracellular environment^15^. As a result, the phospholipid fatty acyl chains in S2 cells have a simple composition and are predominantly composed of SFAs and MUFAs, which can be synthesized *de novo* in most animal cells. Furthermore, cholesterol, which affects the physicochemical properties of the cell membrane, cannot be synthesized *de novo* in insects^16^, resulting in low cholesterol content in *Drosophila* cells^17^. Therefore, phospholipids are expected to play a crucial role in regulating the cell membrane function in S2 cells. Furthermore, *Drosophila* possesses functional homologs of approximately 65% of human disease‑related genes^18^. Given these characteristics, *Drosophila* S2 cells serve as a valuable model for investigating the regulatory mechanisms governing the fatty acid composition of cellular phospholipids and their biological significance.

Glutamine supports numerous cellular functions via its conversion into various metabolites. In mitochondria, glutamine is converted to glutamate via a deamination reaction catalyzed by glutaminase (GLS)^19^. Glutamate is then converted into α-ketoglutarate (α-KG) by glutamate dehydrogenase or mitochondrial aminotransferases, after which α-KG is mobilized into the citric acid cycle or converted to citrate by reductive carboxylation^20,21^. Glutathione (GSH), which is synthesized from glutamate, plays a key role in protecting cells from oxidative stress^20^. Glutamine metabolism is believed to be involved in cellular lipid production. Glutamate-derived α-KG is employed for fatty acid biosynthesis in various cell lines, especially under hypoxic conditions^22^. Moreover, an increased utilization of glutamate-derived α-KG for fatty acid biosynthesis is evident in cells exhibiting mitochondrial dysfunction^23^. In addition to glutamate, ammonia, a byproduct of the GLS reaction, contributes to cellular function by acting as a regulator of intracellular pH. Ammonia generated through glutamine metabolism helps cancer cells mitigate the acid stress resulting from the elevated acidity of the tumor microenvironment^24^. Furthermore, increased ammonia production by GLS prevents the decline in intracellular pH resulting from lysosomal membrane damage in senescent cells^25^. Thus, glutamine metabolism contributes to various cellular functions. However, its role in regulating membrane lipid composition remains unclear.

In this study, we report a correlation between glutamine metabolism and phospholipid composition in S2 cells, with a particular focus on GLS. As part of this analysis, we uncovered a close relationship between the cellular pH and the structural characteristics of cellular phospholipid acyl chains, which may constitute an adaptive mechanism in response to pH fluctuations.

## Methods

### Materials and cell culture

*Drosophila* S2 and Kc167 cells were provided by Dr. Kumiko Ui-Tei and maintained in Schneider’s Drosophila medium, supplemented with 10% fetal bovine serum, 50 units/mL penicillin, and 50 µg/mL streptomycin, at 25 °C. CB-839, BPTES, 968, and CAY10566 were purchased from Cayman Chemicals. GSH reduced ethyl ester (GSH-MEE), dimethyl-α-KG (DM-α-KG), and 5-(N, N-hexamethylene)amiloride (HMA) were obtained from Sigma-Aldrich. Choline-d_9_ was obtained from Cambridge Isotope Laboratories. Cell proliferation was assessed using the Aqueous One Solution Cell Proliferation Assay (Promega).

### Lipid extraction and quantification

Total lipids were extracted from samples using the Bligh & Dyer method^26^. Phosphatidylcholine (PC), phosphatidylethanolamine (PE), phosphatidylserine (PS), phosphatidylinositol (PI), and ceramide phosphoethanolamine (CerPE) were separated from the total lipid extract using two-dimensional thin-layer chromatography^15^. The amount of phospholipids in each spot was determined by inorganic phosphate quantification^27^. The amount of cholesterol was determined using the Wako Cholesterol-E test (Wako).

### Liquid chromatography–mass spectroscopy analysis of lipids

The molecular composition of the phospholipids and triacylglycerol (TG) was analyzed using a Shimadzu LC-30AD high-performance liquid chromatography system coupled with a triple-quadrupole LCMS-8040 mass spectrometer equipped with an electrospray ionization source^28,29^. Separation was performed on a Kinetex C8 column (2.6 μm; 2.1 × 150 mm; Phenomenex) with a binary mobile phase of the following composition: 10 mM ammonium formate in water (mobile phase A) and 10 mM ammonium formate in 2-propanol/acetonitrile/water (45:45:10; v/v/v) (mobile phase B). For phospholipid analysis, the pump controlling the mobile phase B gradient was programmed as follows: an initial isocratic flow of 20% B for 1 min, linear increase to 40% B for 1 min, an increase to 92.5% B using a curved gradient for 23 min, a linear increase to 100% B for 1 min, and holding at 100% B for 4 min. For TG analysis, the pump controlling the mobile phase B gradient was programmed as follows: linear increase from 20% B to 93% B for 5 min, a linear increase to 100% B for 35 min. The total flow rate was 0.3 mL/min, the column temperature was 45 °C, and the sample temperature was 4 °C. The spectrometer parameters were as follows: nebulizer gas flow, 2 L/min; drying gas flow, 15 L/min; interface voltage, 4.5 kV; DL temperature, 250 °C; and heat-block temperature, 400 °C. The transition was [M + H]^+^ → [184.1]^+^ for PC, [M + H]^+^ → [M + H − 141.0]^+^ for PE, and [M + H]^+^ → [M + H − 185.0]^+^ for PS. The fatty acid composition of PC, PE, and PS was determined by product ion scan analysis of [M + HCOO]^−^, [M − H]^−^, and [M − H]^−^ as precursor ions, respectively. TG measurement was conducted by detecting [M + NH_4_]^+^. Measurement of free fatty acids was performed by monitoring the [M – H]⁻ ion species.

### Metabolic labeling of PC with choline-d_9_

To evaluate PC biosynthesis, cells were incubated in culture medium containing 100 µM choline-d_9_, in the presence or absence of CB-839 and ammonia, for up to 24 h. Cellular lipids were extracted using the Bligh & Dyer method and analyzed on an LCMS-8040 system. The mass transition for choline-d_9_-labeled PC was monitored as [M + H]^+^ → 193.15^+^.

### Immunoblotting

The cells were washed with phosphate-buffered saline and lysed in lysis buffer (10 mM Tris-HCl, pH 7.4, 1% Triton X-100, 0.1% SDS, and 1% sodium deoxycholate) containing a 1% protease inhibitor cocktail (Nacalai Tesque). The lysates were centrifuged at 14,000 × *g* for 10 min at 4 °C. The supernatant (20 µL) was mixed with 5 µL sampling buffer 1 (50% sucrose, 50 mM Tris-HCl, pH 8.0, 1% SDS, 5 mM EDTA, and 0.4% bromophenol blue), incubated for 10 min at 50 °C, and then mixed with 25 µL sampling buffer 2 (10% sucrose, 10 mM Tris-HCl, pH 8.0, 0.2% SDS, 1 mM EDTA, 0.08% bromophenol blue, and 60% urea). Samples were electrophoresed on an SDS-polyacrylamide gel and blotted onto a PVDF membrane (Wako) using a Trans-Blot SD Semi-Dry Electrophoretic Transfer Cell (Bio-Rad). Immunoblotting analysis was performed by using anti-DESAT1 antibody^30^ and anti-α-tubulin antibody (MBL, PM054) as a loading control. Bound antibodies were detected with horseradish peroxidase-conjugated anti-rabbit IgG using SuperSignal West Pico (Thermo Fisher Scientific) and LuminoGraph I (ATTO). The band intensity was determined using ImageJ software.

### Real-time PCR

Total RNA was extracted using the TRIzol reagent (Ambion). cDNA was prepared using the ReverTra Ace qPCR RT Master Mix (Toyobo). The level of *DESAT1* and *GLS* mRNA was quantified using StepOne real-time PCR system (Applied Biosystems) with PowerUP SYBR Green Master Mix (Thermo Fisher Scientific) and specific primers (for DESAT1, 5’-ATGGTCACCTCTGCCAAGTG-3’ and 5’-TTGGCCTTGTAGGAGCGATG-3’; for GLS, 5’-CAAACAGCTGCAGCAGAAAAT-3’ and 5’-CTCGCGATGGAGCTTGG-3’; for Rp49, 5’-ATACAGGCCCAAGATCGTGAAG-3’ and 5’-ACGCACTCTGTTGTCGATAC-3’), and quantified using the 2^− ΔΔCt^ method.

### RNA sequencing

Total RNA was extracted using the NucloSpin RNA kit (Takara). Library preparation was performed using an Optimal Dual-mode mRNA Library Prep Kit (BGI). Paired-end sequencing with a read length of 150 bases was performed using a DNBSEQ-G400 platform (BGI).

### Intracellular localization of GLS

The coding sequences of *Drosophila melanogaster* GLS (GH22838; Drosophila Genomics Resource Center Stock #2006) and EGFP were ligated into multiple cloning site of pAc5.1/V5-His-A to construct pAc5.1-GLS-GFP. Cells were transfected with pAc5.1-GLS-GFP using a transfection lipid reagent (Bio-Rad), stained with MitoTracker Deep Red FM (Thermo Fisher Scientific), and observed with confocal microscopy LSM800 (Zeiss).

### Measurement of intracellular and extracellular pH

To measure intracellular pH, the cells were labeled with 2 µM BCECF-AM for 30 min. After labeling, the cells were washed with buffer A (400 mg/L KCl, 8000 mg/L NaCl, 60 mg/L KH_2_PO_4_, 47.9 mg/mL Na_2_HPO_4_, 1000 mg/L D-glucose), suspended in buffer A, and transferred to a black 96-well plate. Fluorescence intensity was measured using a TECAN Infinite 200 plate reader at excitation/emission wavelengths of 440/530 and 500/530 nm. For pH calibration, BCECF-AM-labeled cells were incubated for 10 min in calibration buffer (130 mM KCl, 10 mM NaCl, 1 mM MgSO_4_, and 10 mM Na-MOPS; pH 6.6, 7.0, 7.4, or 7.8) containing 10 µg/mL nigericin, and fluorescence intensity was measured. Extracellular pH (the pH of the culture medium) was measured using a LAQUAtwin-pH-22B pH meter (Horiba).

### Lactate quantification

After cells were cultured with or without CAY10566 for 24 h, the amount of lactate released into the medium was measured using the Lactate Assay Kit WST (DOJINDO). Cellular protein levels were determined using the BCA Protein Assay (Thermo).

### Statistical analysis

Values are presented as means ± S.D. The statistical significance of the differences between mean values was analyzed using a non-paired t-test. Multiple comparisons were performed using Tukey’s test, followed by analysis of variance. Statistical significance was set at *p* < 0.05.

## Results

### Inhibition of GLS activity is correlated with an increase of phospholipids with two double bonds

**Fig. 1:**
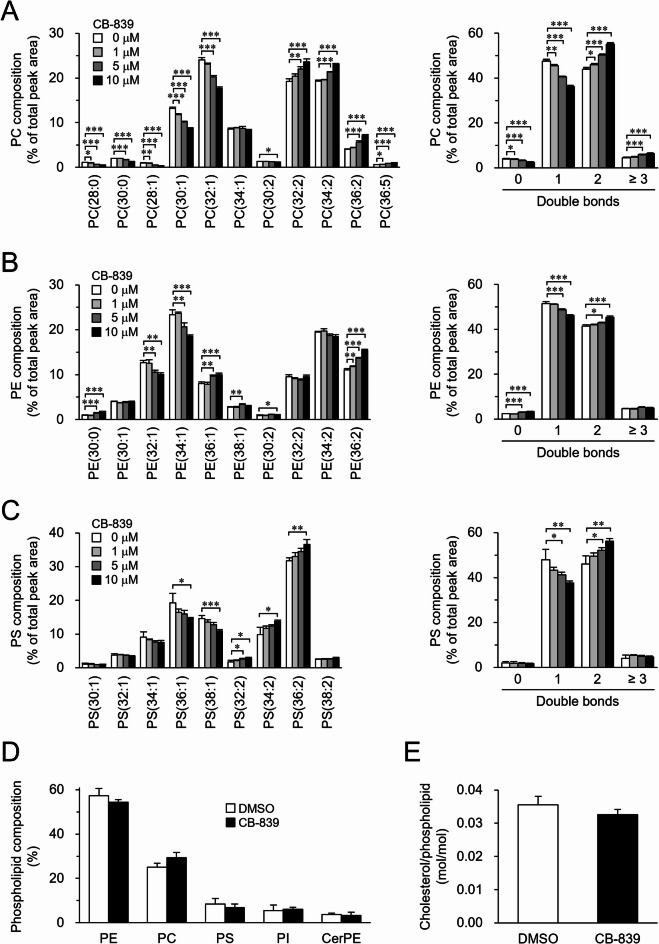
Effect of GLS inhibition on membrane lipid composition. S2 cells were incubated in culture medium in the presence or absence of indicated concentrations (**A**–**C**) or 10 µM (**D**,**E**) of CB-839 for 24 h. The molecular composition of PC (**A**), PE (**B**), and PS (**C**) was analyzed. (**A**–**C**, left) Phospholipid molecules were presented in the format PC(X: Y), PE(X: Y), and PS(X: Y), where X denotes the total number of acyl chain carbons and Y denotes the total number of double bonds in acyl chains. (**A**–**C**, right) Phospholipid molecules were categorized based on the number of double bonds in their acyl chains. The head group composition of phospholipids (**D**) and the cholesterol content (**E**) were analyzed. Mean ± SD (**A**-**D**, *n* = 3; **E**, *n* = 4). *P < 0.05; **P < 0.01; ***P < 0.001.

We first noticed that when S2 cells were treated with the GLS inhibitor CB-839, the molecular composition of PC, PE, and PS was altered (Fig. 1A–C). The proportions of PC molecules with two double bonds, such as PC(32:2), PC(34:2), and PC(36:2), increased in a CB-839 concentration-dependent manner (Fig. 1A, left). In contrast, the proportion of PC molecules with one double bond, such as PC(30:1) and PC(32:1), decreased (Fig. 1A, left). When PC molecules were categorized based on the number of double bonds in their acyl chains, treatment with CB-839 led to a reduction in PC molecules with zero or one double bond, and an increase in those with two or more double bonds (Fig. 1A, right). CB‑839 also increased PC molecules with two double bonds and decreased those with one double bond in another *Drosophila* cell line, Kc167 (Supplementary Fig. S1). In addition, GLS inhibition by CB-839 increased the proportion of PE and PS molecules with two double bonds in S2 cells (Fig. 1B, C). An increase in the proportion of phospholipid molecules with two double bonds was observed in the presence of two additional GLS inhibitors, BPTES (Supplementary Fig. S2) and 968 (Supplementary Fig. S3). These results suggest that GLS is somehow involved in the regulation of the fatty acid composition of cellular phospholipids.

The fatty acid composition of phospholipid molecules, whose levels were altered by GLS inhibition, was examined using product ion scan analysis (Supplementary Fig. S4). Phospholipid molecules with two double bonds, which increased following GLS inhibition, were primarily composed of two MUFAs, such as C16:1 and C18:1 (Supplementary Fig. S4G–L). In contrast, phospholipid molecules with one double bond, which decreased upon GLS inhibition, consisted of one SFA and one MUFA (Supplementary Fig. S4A–F). These results demonstrated that the changes in phospholipid fatty acid composition linked to GLS inhibition were characterized by an increase in MUFA-rich molecules and a decrease in SFA-containing molecules. In contrast to the striking effect on the fatty acyl chain composition of cellular phospholipids, GLS inhibition did not affect the polar head group composition of cellular phospholipids (Fig. 1D) or cellular cholesterol content (Fig. 1E). These findings suggest that GLS selectively regulates the MUFA content in phospholipid acyl chains.

### Consequences of GLS inhibition on phospholipid composition under DESAT1 inhibition

**Fig. 2:**
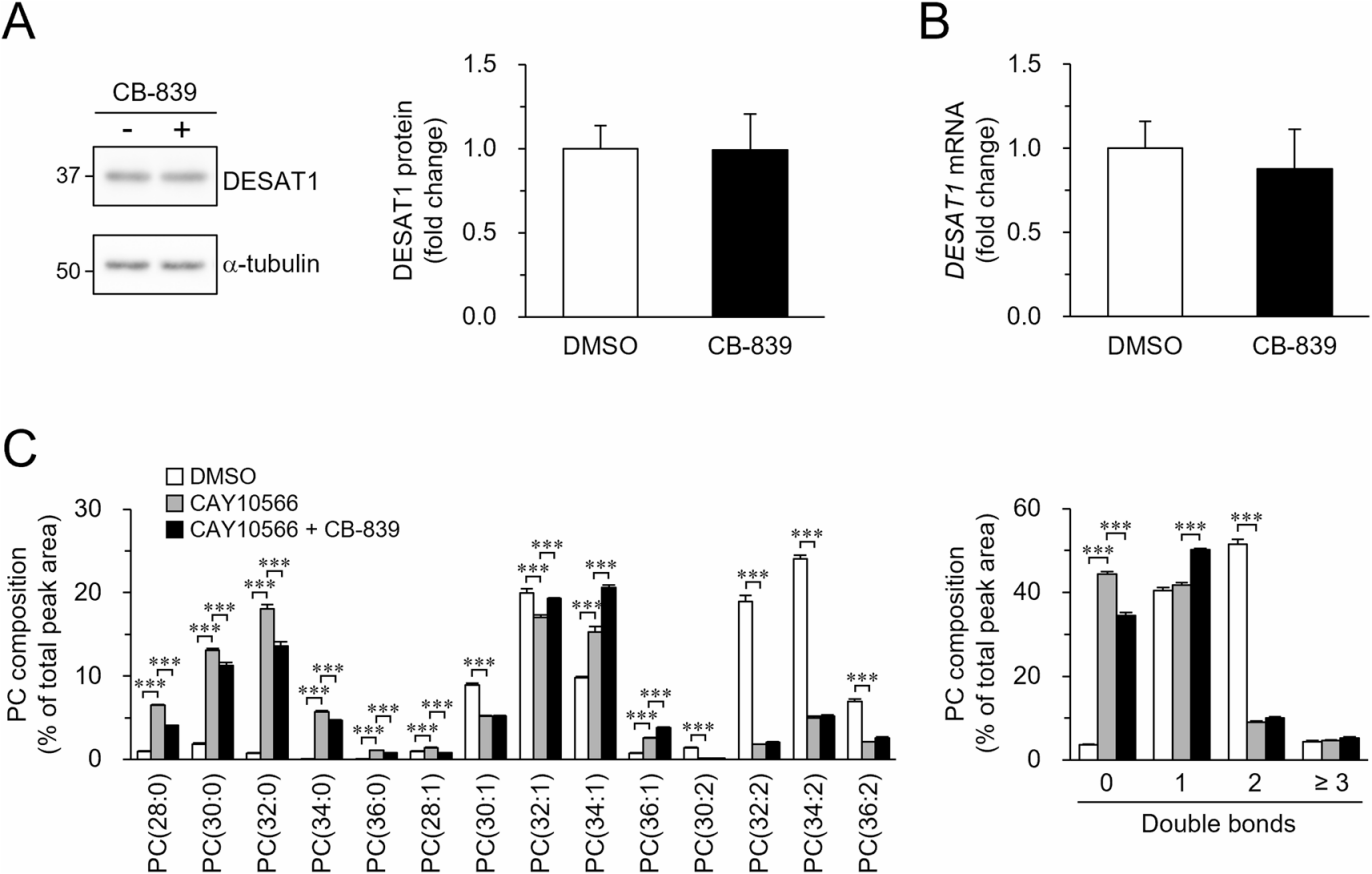
Role of DESAT1 in GLS inhibition-induced phospholipid unsaturation. S2 cells were incubated in culture medium containing DMSO, 10 µM CB-839, and/or 5 µM CAY10566 for 24 h. (**A**) The amounts of DESAT1 and α-tubulin protein were detected with specific antibodies. Numbers on the left of the panels indicate the molecular weights (kDa) of size markers. Full-length blots were shown in Supplementary Fig. S5. (**B**) The amount of DESAT1 mRNA was determined by RT-PCR. (**C**) The molecular composition of PC was analyzed. (**C**, left) PC molecules were presented in the format PC(X: Y), where X denotes the total number of acyl chain carbons and Y denotes the total number of double bonds in acyl chains. (**C**, right) PC molecules were categorized based on the number of double bonds in their acyl chains. Mean ± SD (*n* = 3). ***P < 0.001.

In S2 cells, DESAT1 is the sole Δ9-fatty acid desaturase responsible for the production of MUFAs^7,14^. Thus, changes in DESAT1 expression affect the MUFA content in the fatty acyl chains of cellular phospholipids^7^. Moreover, DESAT1 expression is dynamically regulated at both the transcriptional and post-translational levels^30,31^. Therefore, we investigated whether GLS inhibition leads to changes in DESAT1 expression that could be responsible for the increase in MUFA-rich phospholipids. Contrary to expectations, GLS inhibition did not significantly alter the protein or mRNA levels of DESAT1 (Fig. 2A, B; Supplementary Fig. S5), suggesting that the observed increase in MUFA levels was not due to upregulation of DESAT1 expression.

Therefore, we evaluated whether DESAT1 activity itself was required for the regulation of phospholipid acyl chain composition under GLS-inhibited conditions. Inhibition of DESAT1 by treatment with CAY10566 restricted the supply of MUFAs and consequently altered the phospholipid unsaturation profile, reducing the proportion of phospholipid molecules containing two double bonds and increasing the proportion of those lacking double bonds (Fig. 2C; Supplementary Fig. S6). Under DESAT1-inhibited conditions, treatment with a GLS inhibitor resulted in an increase in PC molecules with one double bond and a decrease in those with no double bonds (Fig. 2C). Furthermore, GLS inhibition increased the proportion of PE and PS molecules with two double bonds, even in the presence of DESAT1 inhibitor (Supplementary Fig. S6). These results indicate that even when cellular MUFA production was reduced by DESAT1 inhibition, cells attempted to enhance the unsaturation of phospholipid acyl chains under GLS-inhibited conditions.

### GLS inhibition selectively promotes the production of MUFA-rich species

**Fig. 3:**
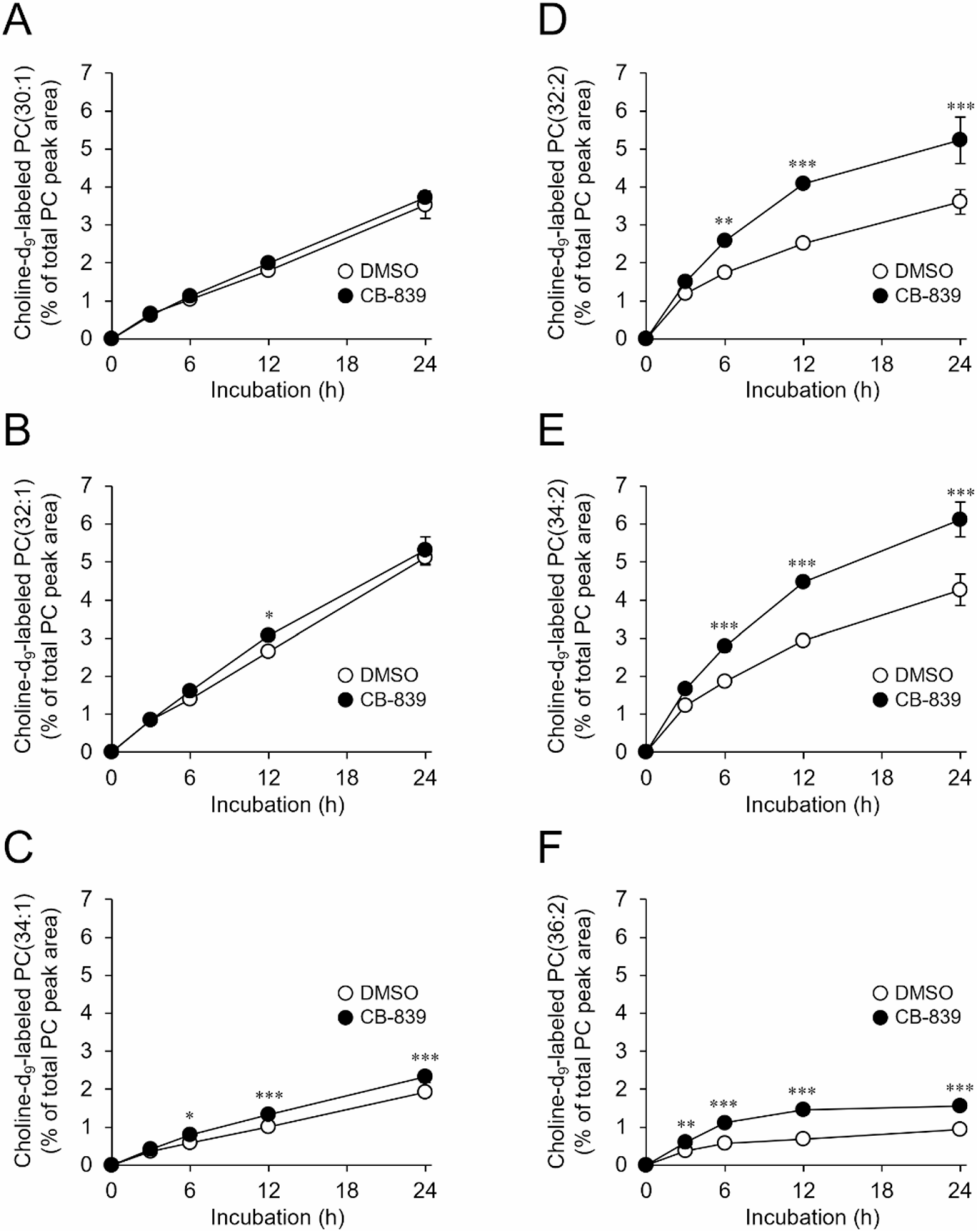
Effect of GLS inhibition on phospholipid biosynthesis. S2 cells were incubated in culture medium containing DMSO or 10 µM CB-839 in the presence of 100 µM choline-d_9_ for up to 24 h. The proportion of choline-d_9_-labeled PC(30:1) (**A**), PC(32:1) (**B**), PC(34:1) (**C**), PC(32:2) (**D**), PC(34:2) (**E**), and PC(36:2) (**F**) in total PC was analyzed. Mean ± SD (*n* = 3). *P < 0.05; **P < 0.01; ***P < 0.001.

In order to determine the effects of GLS inhibition on the expression of genes involved in phospholipid biosynthesis, we carried out RNA sequencing. However, CB-839 treatment caused no apparent changes in the expression of genes encoding protein involved in phospholipid biosynthesis, suggesting that GLS inhibition-induced alterations in phospholipid composition were unlikely to stem from changes in the expression of specific genes (Supplementary Fig. S7). Therefore, we then measured the levels of *de novo*–synthesized PC molecules using metabolic labeling with deuterium-labeled choline (choline-d_9_), which serves as a precursor for PC biosynthesis^32^(Fig. 3). The biosynthesis of PC molecules with two double bonds, PC(32:2), PC(34:2), and PC(36:2), was markedly enhanced by CB-839 treatment, resulting in 1.44-fold, 1.43-fold, and 1.65-fold increases in their levels, respectively, after 24 h of CB-839 exposure (Fig. 3D–F). In contrast, the effect of GLS inhibition on the biosynthesis of PC molecules with one double bond, PC(30:1), PC(32:1), and PC(34:1), was relatively modest, with fold changes of 1.05, 1.03, and 1.21, respectively, under the same treatment conditions (Fig. 3A–C). Triacylglycerol (TG) molecules, which can be synthesized from diacylglycerol (DG)^2^, also showed increased unsaturation upon CB-839 treatment (Supplementary Fig. S8), indicating that the rise in MUFA‑containing phospholipids following GLS inhibition may result from enhanced incorporation of MUFAs into DG, the precursor for phospholipid biosynthesis. Collectively, these results demonstrated that GLS inhibition altered the fatty acid composition of PC molecules by selectively promoting the production of MUFA-rich species.

### Impact of GLS reaction-derived compounds on the unsaturation levels of phospholipid acyl chains

**Fig. 4:**
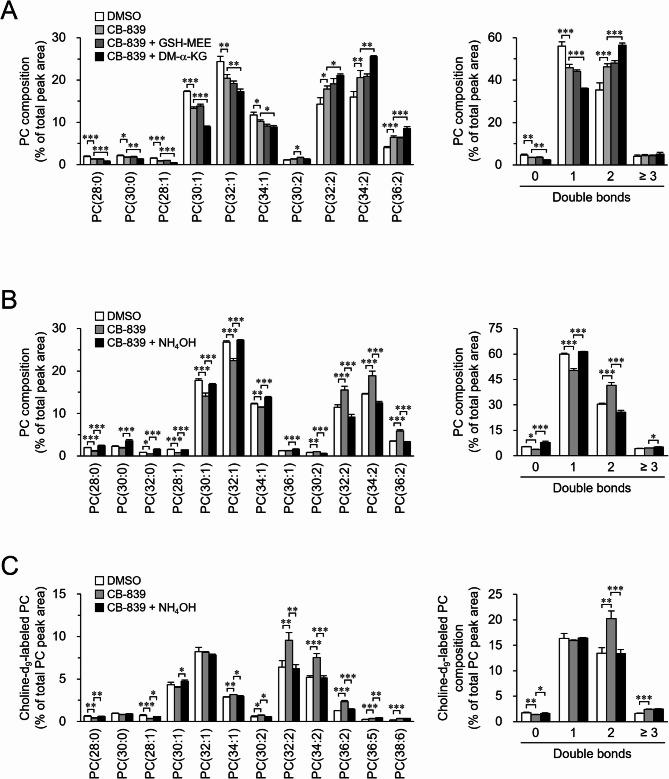
Contribution of GLS reaction products to the regulation of phospholipid composition. (**A**,**B**) S2 cells were incubated in culture medium containing DMSO, 10 µM CB-839, 2 mM GSH-MEE, 7 mM DM-α-KG, and/or 5 mM NH_4_OH for 24 h. The molecular composition of PC was analyzed. (**A**,**B**, left) PC molecules were presented in the format PC(X: Y), where X denotes the total number of acyl chain carbons and Y denotes the total number of double bonds in acyl chains. (**A**,**B**, right) PC molecules were categorized based on the number of double bonds in their acyl chains. (**C**) S2 cells were incubated in culture medium containing DMSO, 10 µM CB-839, and/or 5 mM NH_4_OH in the presence of 100 µM choline-d_9_ for 24 h. (**C**, left) The proportion of choline-d_9_-labeled PC in total PC was analyzed. (**C**, right) Choline-d_9_-labeled PC molecules were categorized based on the number of double bonds in their acyl chains. Mean ± SD (*n* = 3). *P < 0.05; **P < 0.01; ***P < 0.001.

GLS contributes to various cellular functions by metabolizing glutamine in the mitochondria^20^. Given that *Drosophila* GLS is localized to the mitochondria in S2 cells (Supplementary Fig. S9), GLS likely plays a similar role in glutamine metabolism in *Drosophila* as that observed in mammals. Therefore, we examined whether supplementation with cell-permeable derivatives of GSH or α-KG could reverse the changes in the unsaturation levels of cellular phospholipids under GLS inhibition (Fig. 4A; Supplementary Fig. S10A, B). The addition of GSH-MEE did not rescue the CB-839-induced changes in the fatty acid composition of cellular phospholipids. The addition of DM-α-KG rather promoted the increase in the proportion of phospholipid molecules with two double bonds in GLS-inhibited cells. These results indicate that the effects of GLS inhibition on cellular phospholipid unsaturation are not linked to GSH synthesized from glutamate. In contrast, because the addition of α-KG—which can be synthesized from glutamate—increased the proportion of phospholipids with two double bonds in GLS-inhibited cells, it is likely that this metabolite plays a role in this process.

Ammonia is also produced when glutamine is converted to glutamate by the action of GLS. Therefore, we assessed the contribution of ammonia production to the regulation of the cellular phospholipid acyl chain composition (Fig. 4B; Supplementary Fig. S10C, D). The addition of 5 mM ammonia, which has been reported to rescue the effects associated with impaired ammonia production following GLS inhibition in mammalian cells^25^, restored the GLS inhibition-induced increase in the proportion of phospholipid molecules with two double bonds in S2 cells. Furthermore, in GLS-inhibited S2 cells, the addition of ammonia suppressed the *de novo* synthesis of PC molecules with two double bonds but not those with one double bond (Fig. 4C). These results demonstrate that ammonia produced by GLS is involved in regulating the unsaturation levels of cellular phospholipid acyl chains.

### Unsaturation of phospholipid acyl chains in response to changes in extracellular or intracellular pH

**Fig. 5:**
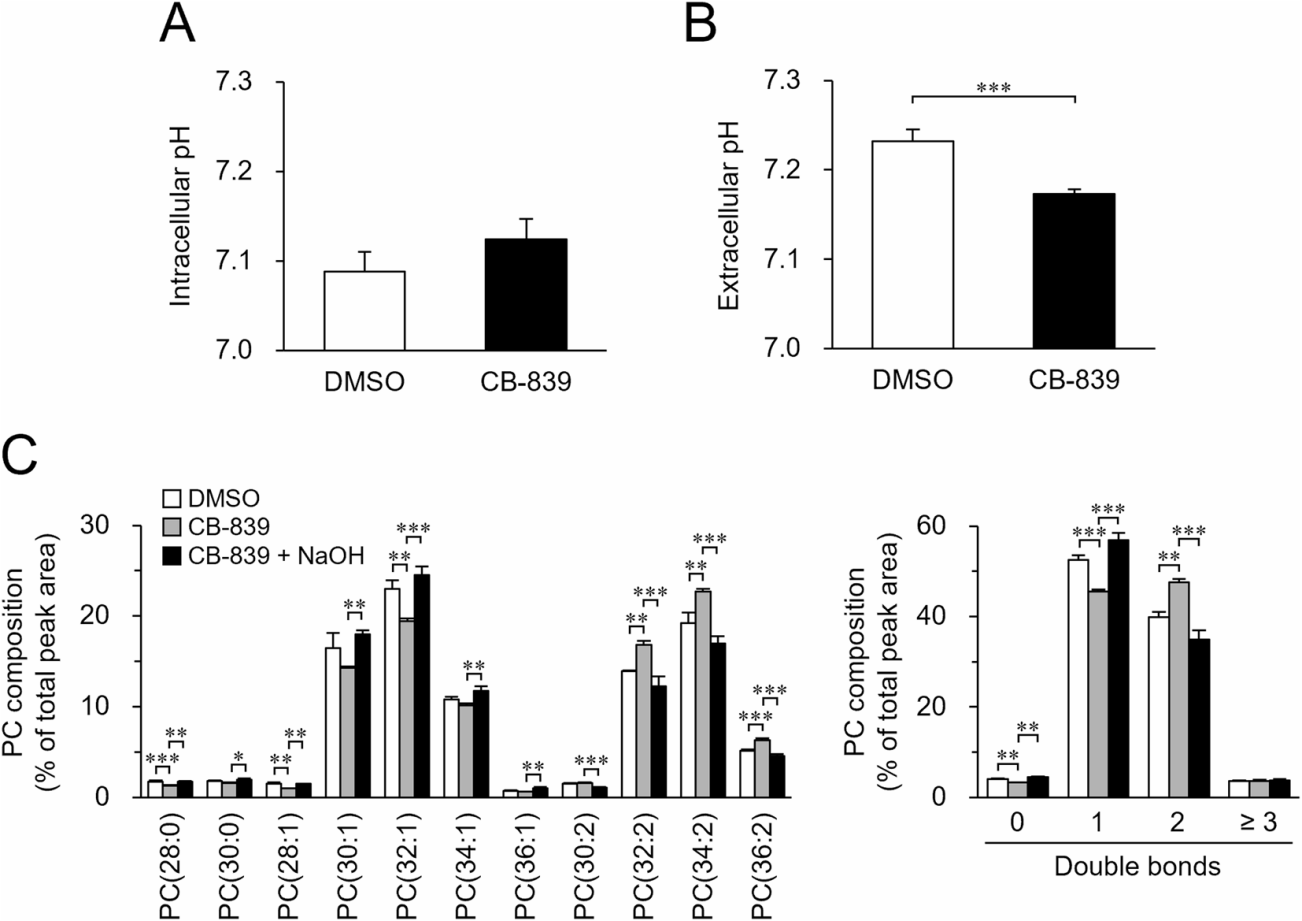
Effect of NaOH on GLS inhibition-induced phospholipid unsaturation. S2 cells were incubated in culture medium containing DMSO, 10 µM CB-839, and/or 3 mM NaOH for 24 h. Intracellular pH (**A**) and extracellular pH (**B**) were measured. (**C**) The molecular composition of PC was analyzed. (**C**, left) PC molecules were presented in the format PC(X: Y), where X denotes the total number of acyl chain carbons and Y denotes the total number of double bonds in acyl chains. (**C**, right) PC molecules were categorized based on the number of double bonds in their acyl chains. Mean ± SD (**A**, **C**, *n* = 3; **B**, *n* = 6). *P < 0.05; **P < 0.01; ***P < 0.001.

Since ammonia produced by GLS is known to regulate intracellular pH^24,25^, we investigated whether ammonia production affected the fatty acid composition of cellular phospholipids through pH modulation. Although CB-839 did not significantly alter intracellular pH in S2 cells, it caused a significant decrease in extracellular pH (Fig. 5A, B). To evaluate whether GLS inhibition affects phospholipid fatty acid composition via pH changes, we used NaOH instead of ammonia. The addition of 3 mM NaOH altered the pH of the culture medium to an extent similar to the addition of 5 mM ammonia (Table 1). The increased proportion of phospholipids with two double bonds in GLS-inhibited cells was reversed by the addition of 3 mM NaOH to the culture medium (Fig. 5C; Supplementary Fig. S11), suggesting that the basic properties of ammonia contribute to the regulation of phospholipid composition. Furthermore, although cell proliferation was suppressed in a CB‑839 dose‑dependent manner, this effect was rescued by the addition of ammonia or NaOH (Supplementary Fig. S12A), suggesting that GLS inhibition in S2 cells may, at least in part, exert its effects through changes in pH.

**Fig. 6:**
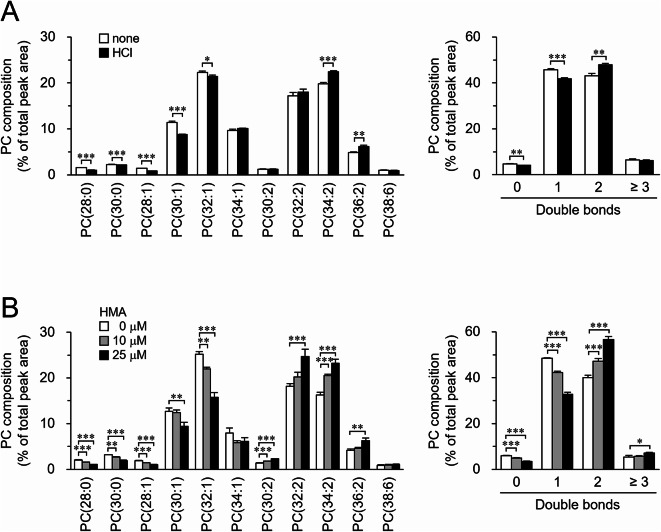
Effect of acidic stress on phospholipid composition. (**A**) S2 cells were incubated in culture medium in the presence or absence of 4 mM HCl for 24 h. (**B**) S2 cells were incubated in culture medium containing indicated concentrations of HMA for 24 h. (**A**,**B**) The molecular composition of PC was analyzed. (A, B, left) PC molecules were presented in the format PC(X: Y), where X denotes the total number of acyl chain carbons and Y denotes the total number of double bonds in acyl chains. (**A**,**B**, right) PC molecules were categorized based on the number of double bonds in their acyl chains. Mean ± SD (*n* = 3). *P < 0.05; **P < 0.01; ***P < 0.001.

Therefore, we examined whether the fatty acid composition of cellular phospholipids could be modulated by altering the pH to which cells are exposed through approaches other than GLS inhibition. Addition of 4 mM HCl lowered the pH of the culture medium (Table 1) and increased the proportion of phospholipid molecules with two double bonds (Fig. 6A; Supplementary Fig. S13A, B). Furthermore, a Na^+^/H^+^ exchanger inhibitor (HMA), which suppresses the excretion of cellular H^+^ into the extracellular environment, decreased intracellular pH (Supplementary Fig. S14) and increased the proportion of phospholipid molecules with two double bonds (Fig. 6B; Supplementary Fig. S13C, D). HMA also increased PC molecules with two double bonds and decreased those with one double bond in Kc167 cells (Supplementary Fig. S1). Similar to the effect of CB‑839, treatment with HCl or HMA also suppressed cell proliferation (Supplementary Fig. S12B, C). These results suggest that the fatty acid composition of cellular phospholipids could be altered by lowering the extracellular or intracellular pH.

### Role of phospholipid unsaturation in cellular adaptation to pH fluctuations

**Fig. 7:**
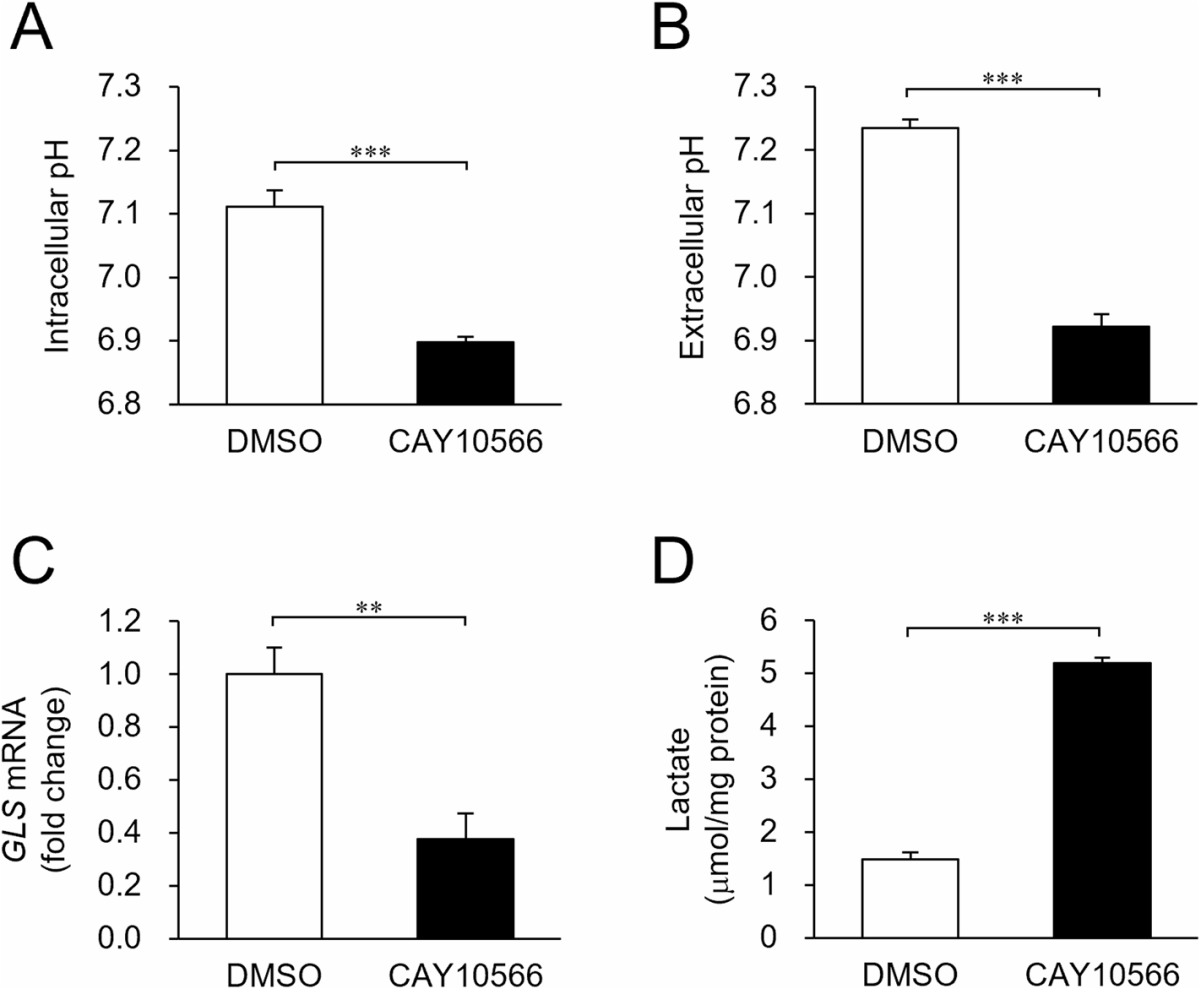
Contribution of MUFA-rich phospholipids to maintenance of pH homeostasis. S2 cells were incubated in culture medium in the presence or absence of 5 µM CAY10566 for 24 h. Intracellular pH (**A**) and extracellular pH (**B**) were measured. (**C**) The amount of *GLS* mRNA was determined by RT-PCR. (**D**) Lactate production was measured. Mean ± SD (**A**, **C**, **D**, *n* = 3; **B**, *n* = 6). **P < 0.01; ***P < 0.001.

Finally, we examined the impact of membrane lipid unsaturation on adaptation to pH fluctuations. When the cellular MUFA levels were reduced by inhibiting DESAT1 (Fig. 2C; Supplementary Fig. S6), intracellular pH decreased from 7.11 ± 0.03 to 6.90 ± 0.01 (Fig. 7A) and extracellular pH decreased from 7.24 ± 0.01 to 6.92 ± 0.02 (Fig. 7B). We also determined whether the degree of phospholipid unsaturation affects GLS expression. As shown in Fig. 7C, DESAT1 inhibition significantly decreased GLS expression. Furthermore, as MUFA content in phospholipid acyl chains affects mitochondrial oxidative phosphorylation^7^, lactate production was also altered by DESAT1 inhibition (Fig. 7D). Taken together, our findings indicate that not only does cellular pH influence phospholipid composition, but changes in phospholipid composition also affect cellular pH. This suggests that cells respond to pH fluctuations by modulating the unsaturation levels of phospholipid acyl chains, which in turn contributes to the maintenance of cellular pH homeostasis.

## Discussion

Our study revealed that the inhibition of GLS, a key enzyme that catalyzes the deamination of glutamine into glutamate, yielding ammonia, leads to an increase in the unsaturation of fatty acids in phospholipids and TG, which appears to be linked to changes in extracellular or intracellular pH. Interestingly, supplementation of the medium with ammonia or NaOH, which leads to an increase in extracellular pH, suppressed the accumulation of MUFA-rich phospholipids under GLS inhibition (Fig. 4B; Supplementary Fig. S10C, D; Fig. 5C; Supplementary Fig. S11). In contrast, lowering the pH of the medium with HCl or reducing the intracellular pH by inhibiting the Na^+^/H^+^ exchanger led to an increase in MUFA-rich phospholipids (Fig. 6; Supplementary Fig. S13), which was similar to that observed under GLS inhibition. Consistently, GLS inhibition decreased the extracellular pH of S2 cells (Fig. 5B). Taken together, these results suggest that the increase in the unsaturation of fatty acids present in phospholipids following GLS inhibition is correlated with the acidification of the extracellular or intracellular space. This finding suggests the existence of a process that regulates the fatty acid composition of cellular phospholipids in response to pH fluctuations. In that respect, it is noteworthy that supplementation of the culture medium with α-KG, a metabolite derived from glutamate, further increased the proportion of phospholipid molecules containing two MUFAs in GLS-inhibited cells (Fig. 4A; Supplementary Fig. S10A, B). This might be because α‑KG is an acidic molecule. However, we cannot rule out that α‑KG, as a key metabolite of the citric acid cycle, could generate citrate, which could then be converted by ATP‑citrate lyase into oxaloacetate and acetyl‑CoA, substrates that can be used for fatty‑acid biosynthesis^22,23^. In contrast, supplementation of cells with GSH, an antioxidant derived from glutamate, had no apparent impact on the phospholipid composition altered by GLS inhibition (Fig. 4A; Supplementary Fig. S10A, B). This suggests that GSH‑mediated redox regulation does not play a role in controlling phospholipid unsaturation levels.

How does acidification enhances unsaturation in fatty acid of phospholipids? The only known enzyme responsible for the introduction of double bonds into fatty acids in S2 cells is DESAT1, because its depletion caused an abolishment of MUFA production^7^. However, the expression level of DESAT1 was unaffected by GLS inhibition (Fig. 2A, B). Moreover, even when DESAT1 was inhibited and MUFA availability markedly reduced, MUFA-containing phospholipids increased under GLS-inhibited conditions (Fig. 2C; Supplementary Fig. S6). These findings suggest that the increase in MUFA content within phospholipid acyl chains upon GLS inhibition is not merely attributable to enhanced MUFA production by DESAT1. Although S2 cells do not efficiently utilize lipoprotein-derived fatty acids for phospholipid biosynthsis^15^, they can use extracellular free fatty acid^30^, such as C16:1 and C18:1, present in the serum used for cell culture (Supplementary Fig. S15). Thus, further studies using lipid‑depleted medium would lead to a better understanding of the contributions of DESAT1 and biosynthesized MUFAs to phospholipid biosynthesis under GLS‑inhibited and pH‑fluctuating conditions. Molecular composition analysis and metabolic labeling experiments revealed the selective increase of MUFA‑rich phospholipids and TG levels under GLS inhibition (Figs. 1 and 3; Supplementary Fig. S8). However, GLS inhibition did not affect the mRNA expression of genes encoding the enzymes involved in the biosynthesis of these molecules (Supplementary Fig. S7), suggesting that the observed increase in phospholipid unsaturation was unlikely to be primarily driven by transcriptional changes. Alternatively, because lysophospholipid acyltransferases—which incorporate fatty acyl chains into phospholipids—exhibit preferences for the structure of their fatty acyl chain substrates^33^, and because mouse LPCAT2, a member of this enzyme family, has been reported to be regulated by serine phosphorylation^34^, post‑translational regulation of biosynthetic enzymes may be involved in the lipid remodeling linked to GLS inhibition. In addition, fatty acids have diverse metabolic fates—not only their incorporation into phospholipids and TG, but also protein acylation, sphingolipid biosynthesis, sterol esterification, conversion to signaling molecules, degradation via β-oxidation, and even their release into the extracellular space^2,35–39^. Thus, changes in the intracellular flux of fatty acids with specific structural features may contribute to the enhanced unsaturation of phospholipid acyl chains observed in GLS-inhibited cells. Since these processes might occur downstream of the cellular pH‑sensing machinery, further studies are needed to advance our understanding of how cells sense and adapt to pH fluctuations.

What is the biological relevance of the phospholipid acyl chain unsaturation induced by exposure to an acidic environment? We demonstrated that MUFA content in cellular phospholipids positively correlates with both intracellular and extracellular pH (Fig. 7A, B). This effect may involve mechanisms such as control of GLS expression (Fig. 7C) or regulation of lactate production (Fig. 7D). These underlying processes are thought to be mediated by complex mechanisms, including metabolic pathway rewiring and the impacts on mitochondrial oxidative phosphorylation^7,40^. In addition, controlling the MUFA content may be advantageous for modulating the physicochemical properties of bilayer membranes in response to pH fluctuations. PS contains a carboxyl group with a pK_a_ within the physiological pH range^41^. Thus, the charge state of PS molecules may fluctuate in response to changes in the pH to which cells are exposed. Membrane fluidity influences the ionization of the carboxyl group of PS, and conversely, the ionization state of the carboxyl group of PS affects membrane fluidity^41,42^. Thus, changes in the MUFA content may modulate the charge of phospholipids in a manner that supports cellular adaptation to acidic environments. Given that the fatty acid composition of phospholipids significantly influences the physicochemical properties and functions of cell membranes, the enhanced unsaturation of phospholipid acyl chains may constitute an adaptive response to acidic stress and confer multiple benefits to cells.

Finally, our study revealed that the pH to which cells are exposed and the MUFA content in cellular phospholipids influence each other. Similar reciprocal regulation has been reported in cellular responses to temperature changes. When the ambient temperature decreases, the number of double bonds in the phospholipid acyl chains increases^7,43,44^, and this enhances membrane fluidity, thereby contributing to the maintenance of membrane function^8^. These observations suggest that environmental changes can trigger lipid remodeling that may facilitate cellular adaptation to fluctuating conditions. Taken together, our findings highlight phospholipid remodeling as a pivotal mechanism through which cells adapt to fluctuating cellular conditions, thereby supporting cellular homeostasis.

**Table 1:** The pH of culture medium.

	pH
Culture medium	6.89
Culture medium + 5 mM NH_4_OH	7.28
Culture medium + 3 mM NaOH	7.41
Culture medium + 4 mM HCl	6.36

## Supplementary Information

Below is the link to the electronic supplementary material.

Supplementary Material 1

## Data Availability

All data are contained within the article and supporting information.
