# Supplementary material for: Glutaminase inhibition is correlated with an increase in phospholipid unsaturation, a potential cellular adaptation to pH fluctuations
Source: Sci Rep. 2026 Apr 3;16:15923. doi: 10.1038/s41598-026-45555-5 (PMC13194661; doi:10.1038/s41598-026-45555-5)
Supplement: Supplementary file 1 — Supplementary Material 1 [file 41598_2026_45555_MOESM1_ESM.pdf]

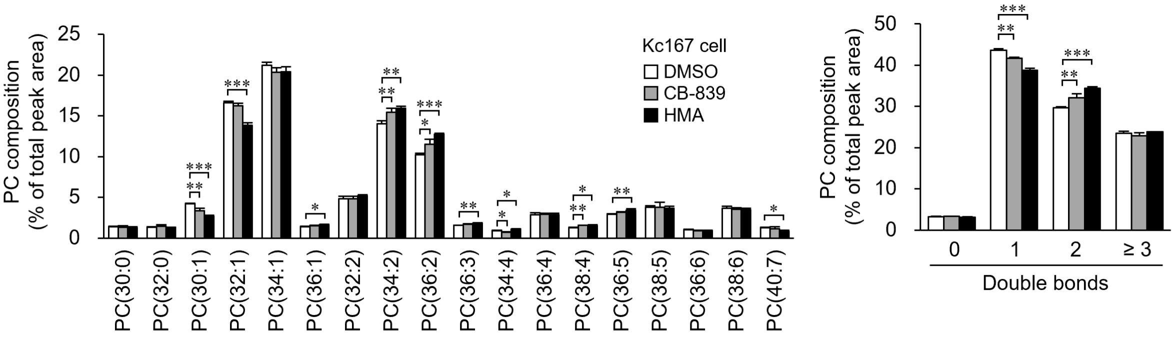

Supplementary Fig. S1. Effect of CB-839 and HMA on PC composition in Kc167 cells. Kc167 cells were incubated in culture medium containing DMSO, 10 μM CB-839, or 25 μM HMA for 24 h. The molecular composition of PC was analyzed. (left) PC molecules were presented in the format PC(X:Y), where X denotes the total number of acyl chain carbons and Y denotes the total number of double bonds in acyl chains. (right) PC molecules were categorized based on the number of double bonds in their acyl chains. Mean ± SD (n = 3). *P < 0.05; **P < 0.01; ***P < 0.001.

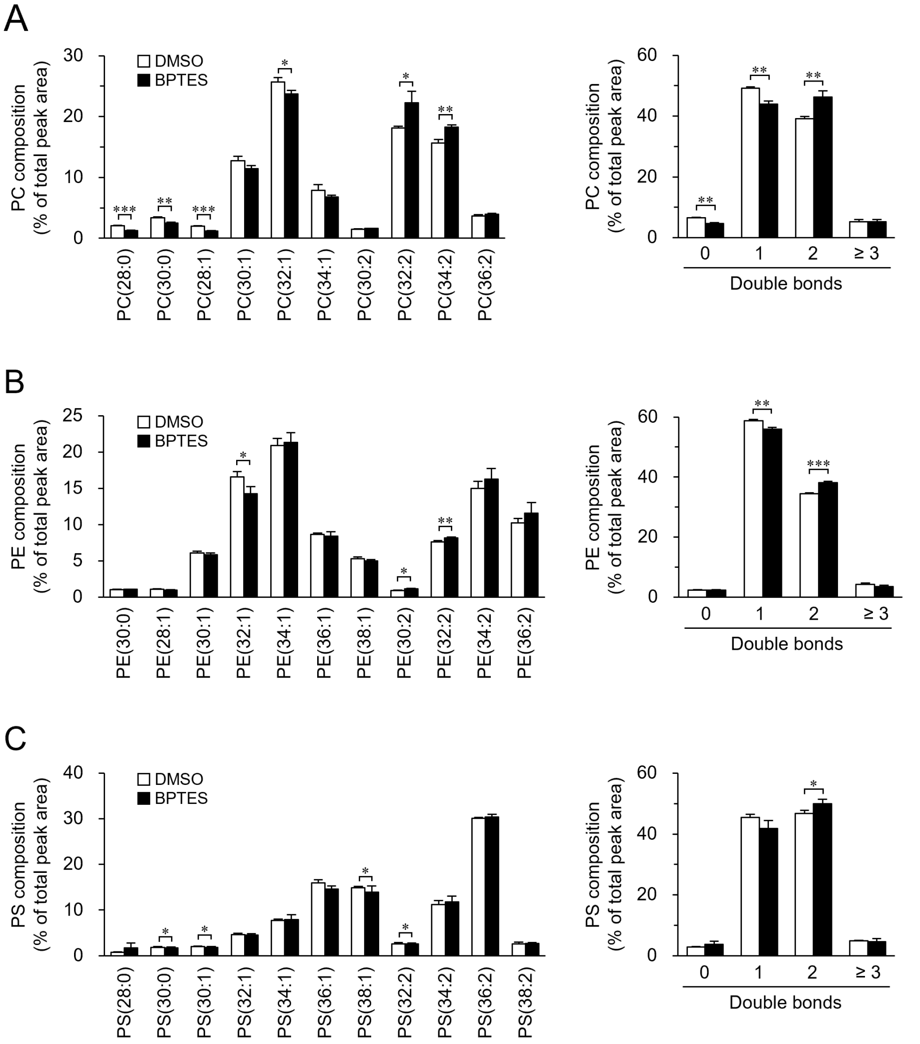

Supplementary Fig. S2. Effect of BPTES on phospholipid composition.

S2 cells were incubated in culture medium in the presence or absence of 10 μM BPTES for 24 h. The molecular composition of PC (*A*), PE (*B*), and PS (*C*) was analyzed. (*A*-*C*, left) Phospholipid molecules were presented in the format PC(X:Y), PE(X:Y), and PS(X:Y), where X denotes the total number of acyl chain carbons and Y denotes the total number of double bonds in acyl chains. (*A*-*C*, right) Phospholipid molecules were categorized based on the number of double bonds in their acyl chains. Mean ± SD (n = 3). *$P < 0.05$; **$P < 0.01$; ***$P < 0.001$.

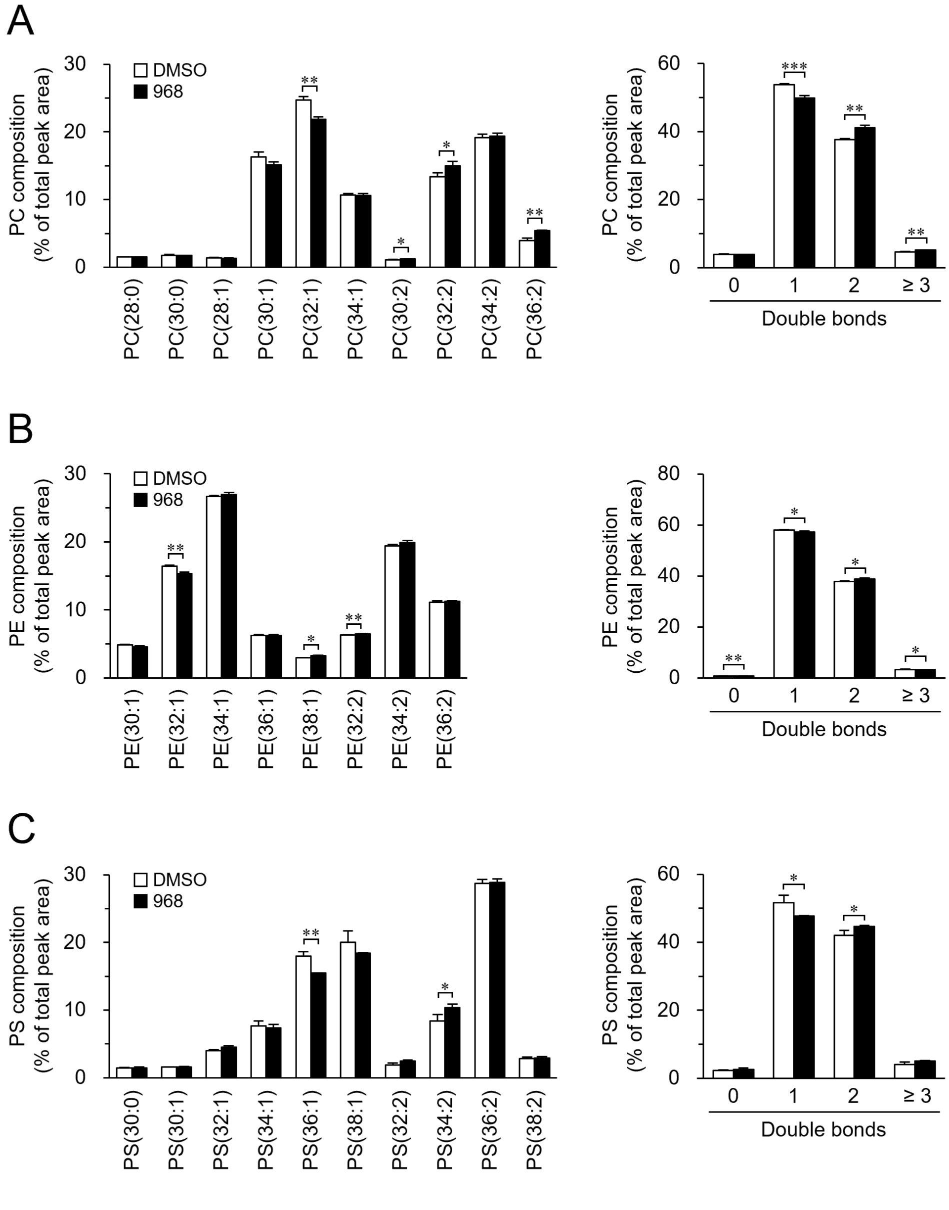

Supplementary Fig. S3. Effect of 968 on phospholipid composition.
S2 cells were incubated in culture medium in the presence or absence of 5 μM 968 for 24 h. The molecular composition of PC (*A*), PE (*B*), and PS (*C*) was analyzed. (*A-C*, left) Phospholipid molecules were presented in the format PC(X:Y), PE(X:Y), and PS(X:Y), where X denotes the total number of acyl chain carbons and Y denotes the total number of double bonds in acyl chains. (*A-C*, right) Phospholipid molecules were categorized based on the number of double bonds in their acyl chains. Mean ± SD ($n = 3$). **P* < 0.05; ***P* < 0.01; ****P* < 0.001.

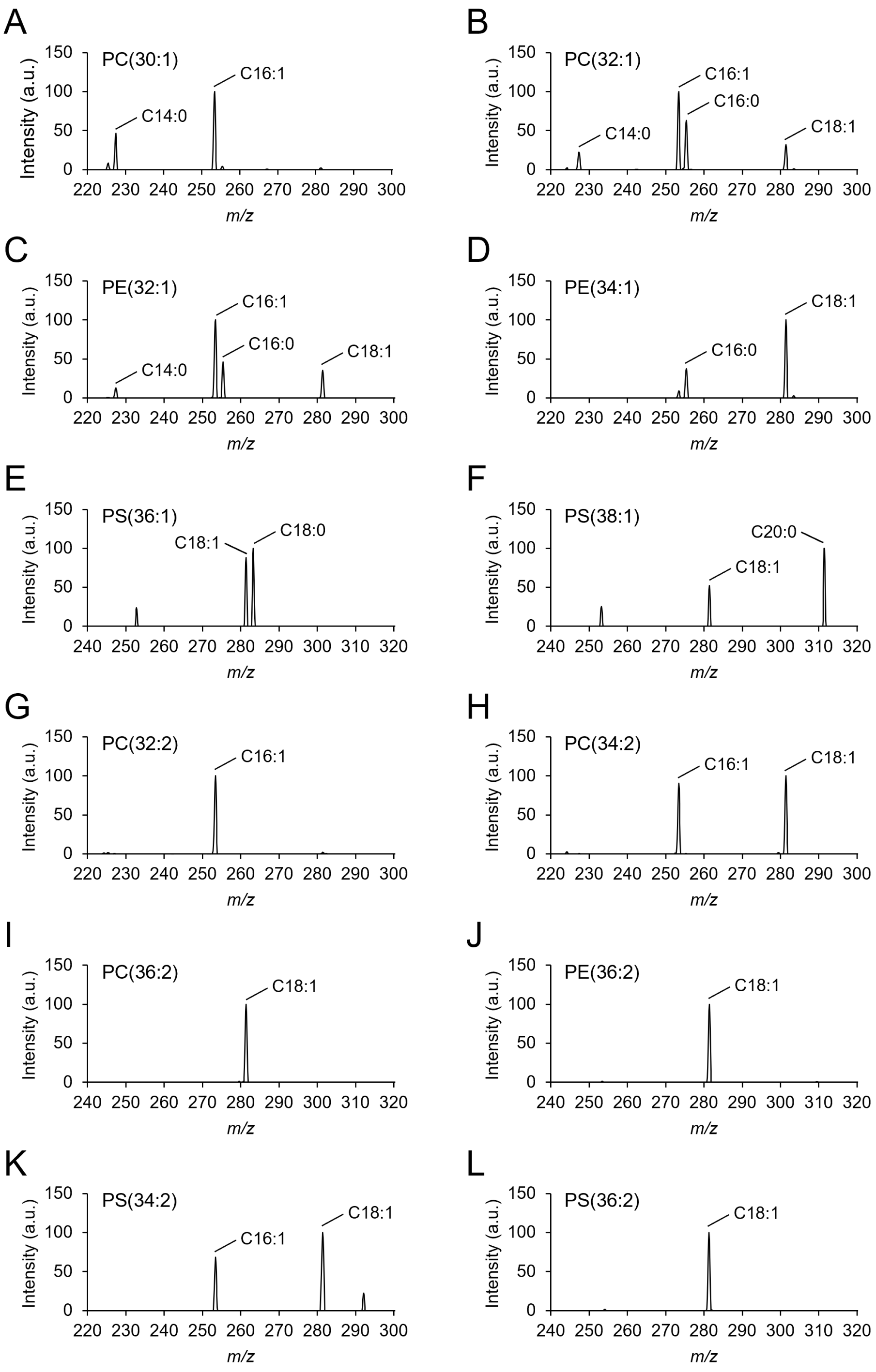

Supplementary Fig. S4. Fatty acid composition of phospholipid molecules.
Product ion scan analysis of PC(30:1) (*A*), PC(32:1) (*B*), PE(32:1) (*C*), PE(34:1) (*D*), PS(36:1) (*E*), PS(38:1) (*F*), PC(32:2) (*G*), PC(34:2) (*H*), PC(36:2) (*I*), PE(36:2) (*J*), PS(34:2) (*K*), and PS(36:2) (*L*) extracted from S2 cells treated with 10 μM CB-839 for 24 h.

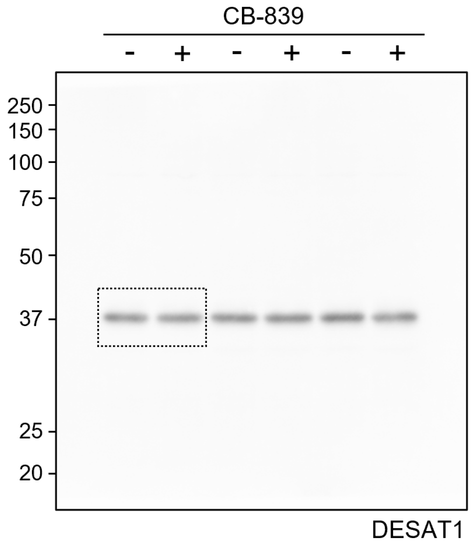

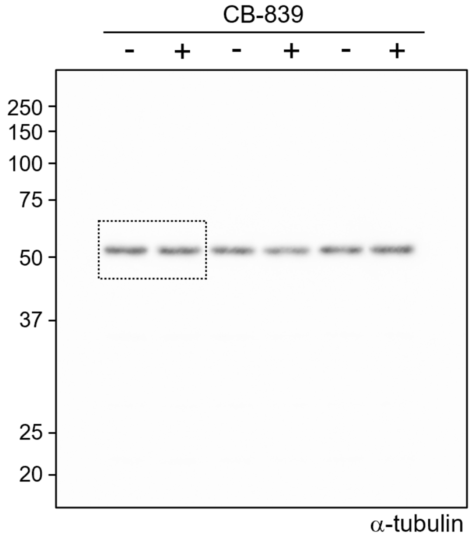

Supplementary Fig. S5. Full-length blots.
The full-length blots corresponding to Fig. 2*A*. Numbers on the left of the panels indicate the molecular weights (kDa) of size markers.

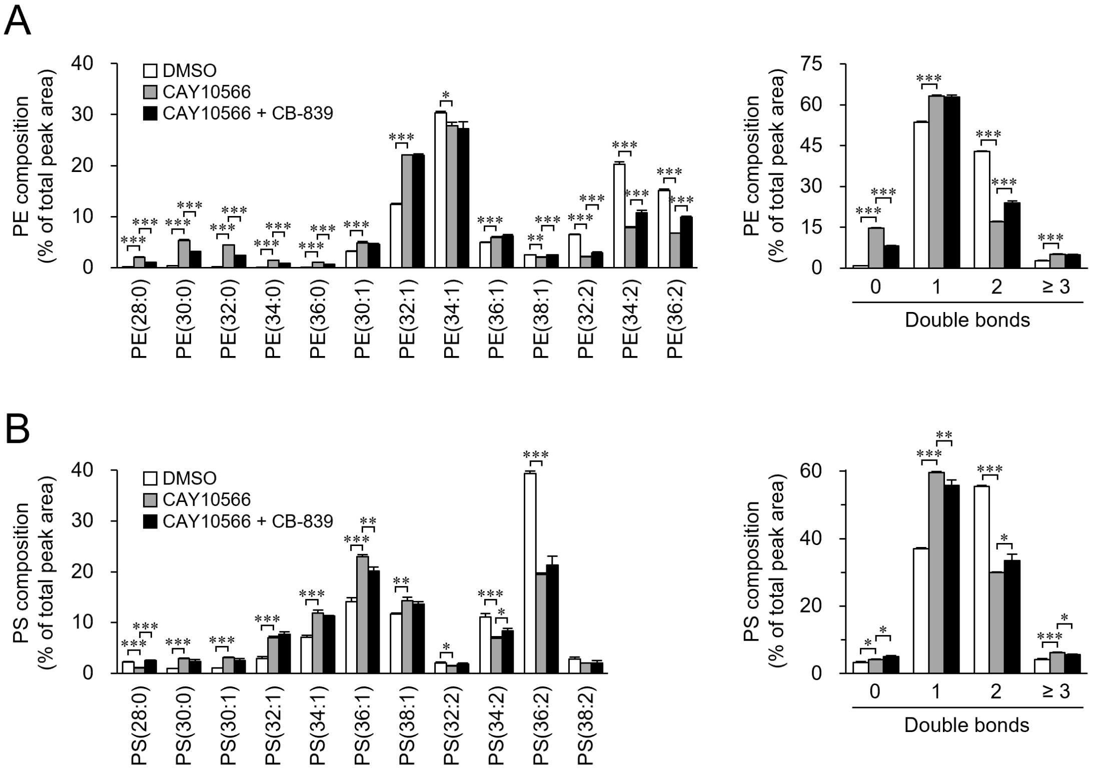

Supplementary Fig. S6. Role of DESAT1 in GLS inhibition-induced unsaturation of PE and PS.

S2 cells were incubated in culture medium containing DMSO, 10 μM CB-839, and/or 5 μM CAY10566 for 24 h. The molecular composition of PE (*A*) and PS (*B*) was analyzed. (*A*,*B*, left) Phospholipid molecules were presented in the format PE(X:Y) and PS(X:Y), where X denotes the total number of acyl chain carbons and Y denotes the total number of double bonds in acyl chains. (*A*,*B*, right) Phospholipid molecules were categorized based on the number of double bonds in their acyl chains. Mean ± SD (n = 3). *$P < 0.05$; **$P < 0.01$; ***$P < 0.001$.

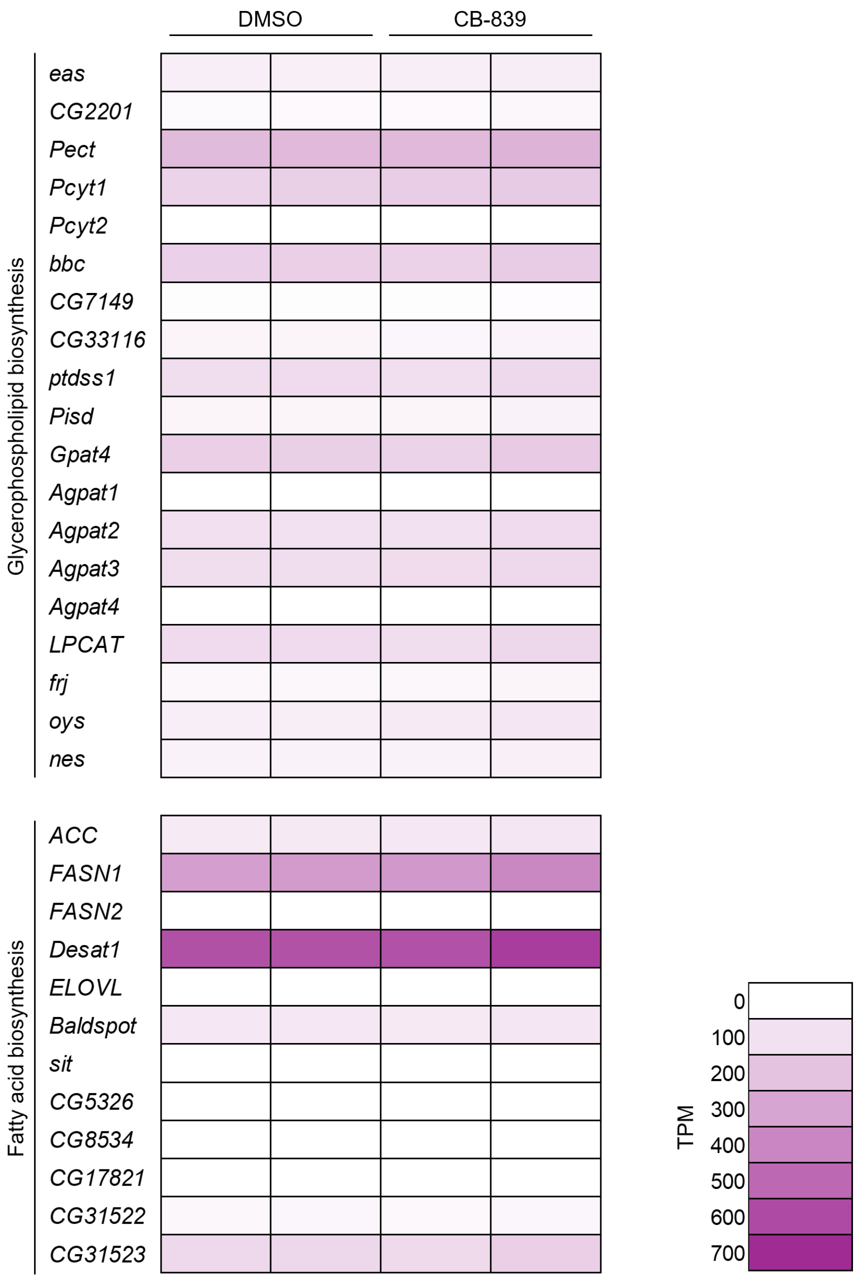

Supplementary Fig. S7. Effect of GLS inhibition on the expression of genes related to phospholipid biosynthesis.
S2 cells were incubated in culture medium in the presence or absence of 10 µM CB-839 for 24 h. Gene expression levels (TPM: transcripts per million) related to the biosynthesis of glycerophospholipids and fatty acids were analyzed by RNA sequencing.

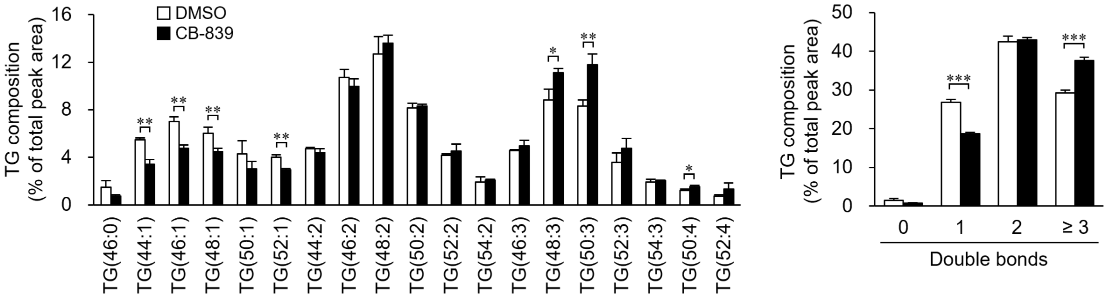

Supplementary Fig. S8. Effect of CB-839 on TG composition.

S2 cells were incubated in culture medium in the presence or absence of 10 μM CB-839 for 24 h. The molecular composition of TG was analyzed. (left) TG molecules were presented in the format TG(X:Y), where X denotes the total number of acyl chain carbons and Y denotes the total number of double bonds in acyl chains. (right) TG molecules were categorized based on the number of double bonds in their acyl chains. Mean ± SD (n = 3). *P < 0.05; **P < 0.01; ***P < 0.001.

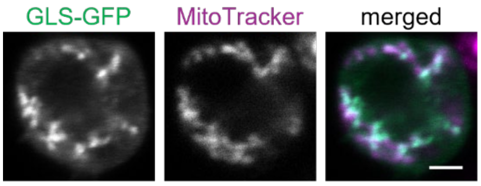

Supplementary Fig. S9. Intracellular localization of GLS.
The localization of GLS-GFP in S2 cells was visualized using confocal microscopy. Scale bar: 2 μm.

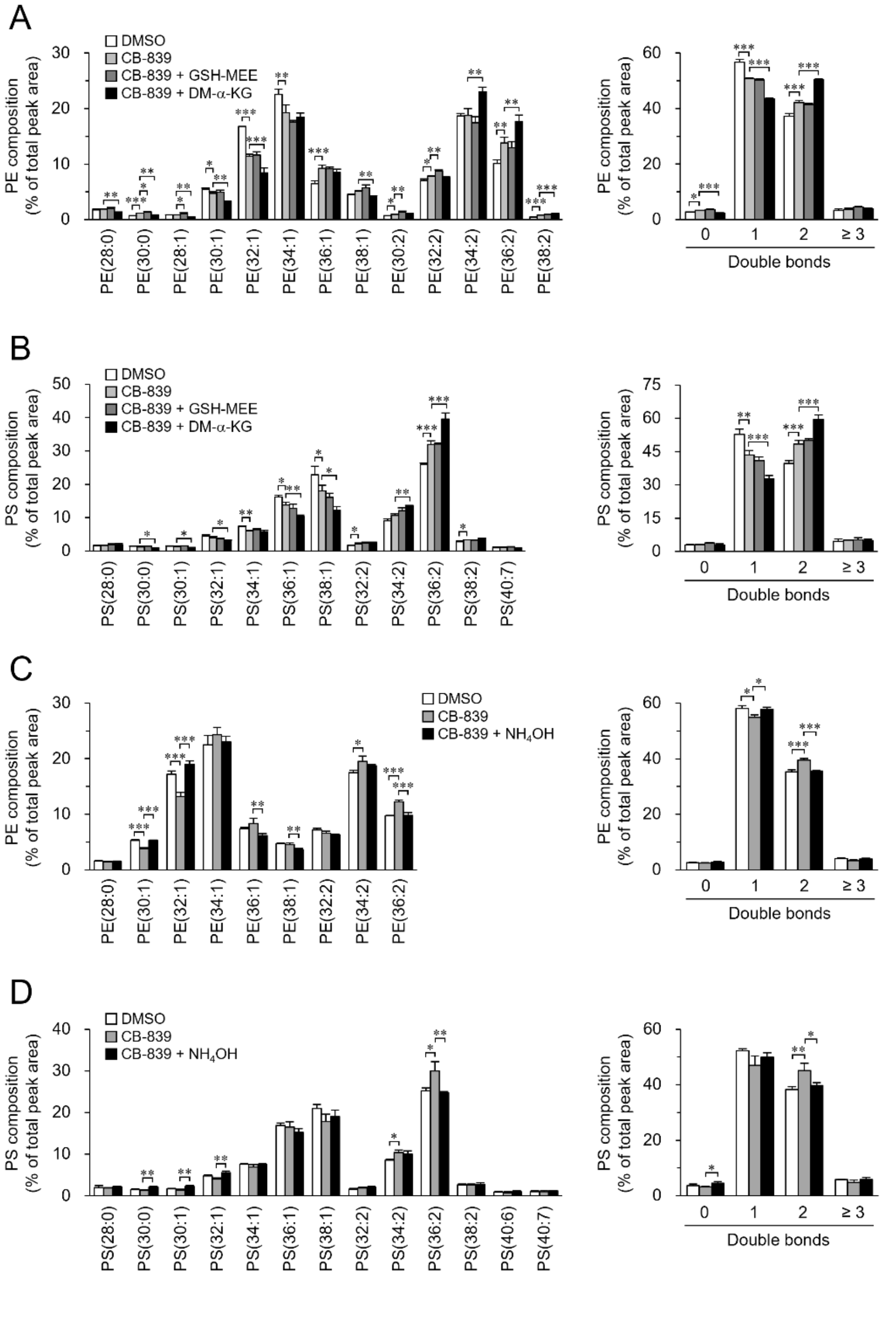

Supplementary Fig. S10. Contribution of GLS reaction products to the regulation of PE and PS composition.

S2 cells were incubated in culture medium containing DMSO, 10 μM CB-839, 2 mM GSH-MEE, 7 mM DM-α-KG, and/or 5 mM NH_4OH for 24 h. The molecular composition of PE (*A*,*C*) and PS (*B*,*D*) was analyzed. (*A-D*, left) Phospholipid molecules were presented in the format PE(X:Y) and PS(X:Y), where X denotes the total number of acyl chain carbons and Y denotes the total number of double bonds in acyl chains. (*A-D*, right) Phospholipid molecules were categorized based on the number of double bonds in their acyl chains. Mean ± SD (*n* = 3). *$P < 0.05$; **$P < 0.01$; ***$P < 0.001$.

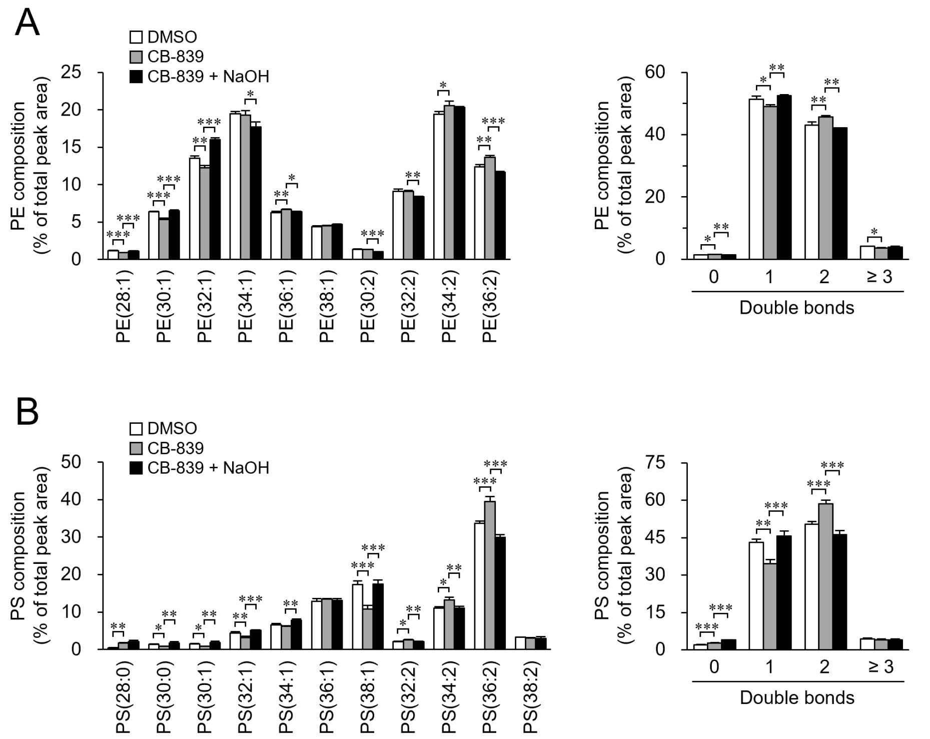

Supplementary Fig. S11. Effect of NaOH on GLS inhibition-induced unsaturation of PE and PS.

S2 cells were incubated in culture medium containing DMSO, 10 μM CB-839, and/or 3 mM NaOH for 24 h. The molecular composition of PE (*A*) and PS (*B*) was analyzed. (*A*,*B*, left) Phospholipid molecules were presented in the format PE(X:Y) and PS(X:Y), where X denotes the total number of acyl chain carbons and Y denotes the total number of double bonds in acyl chains. (*A*,*B*, right) Phospholipid molecules were categorized based on the number of double bonds in their acyl chains. Mean ± SD (n = 3). *$P < 0.05$; **$P < 0.01$; ***$P < 0.001$.

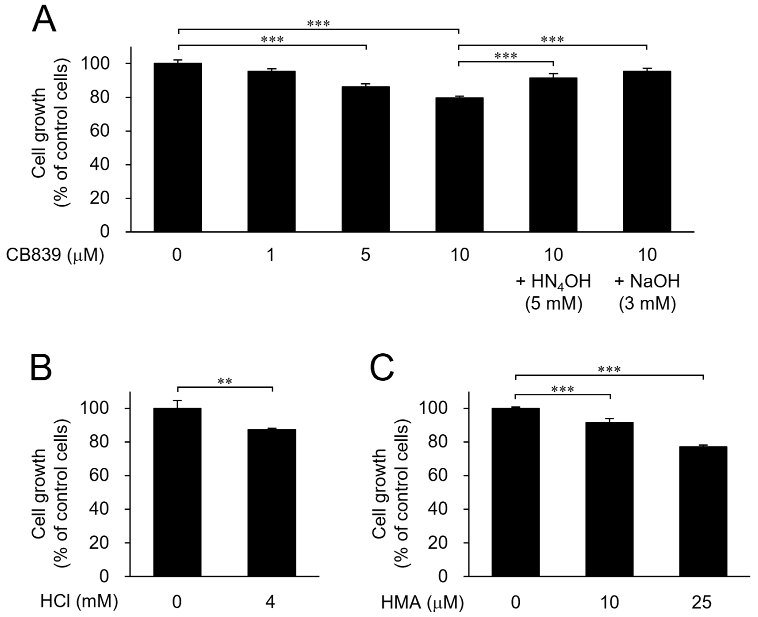

Supplementary Fig. S12. Effect of GLS inhibition and pH manipulations on cell growth. S2 cells were incubated in culture medium containing indicated compounds for 24 h. Cell growth was evaluated using the Aqueous One Solution Cell Proliferation Assay. Mean ± SD ($n = 3$). $^{**}P < 0.01$; $^{***}P < 0.001$.

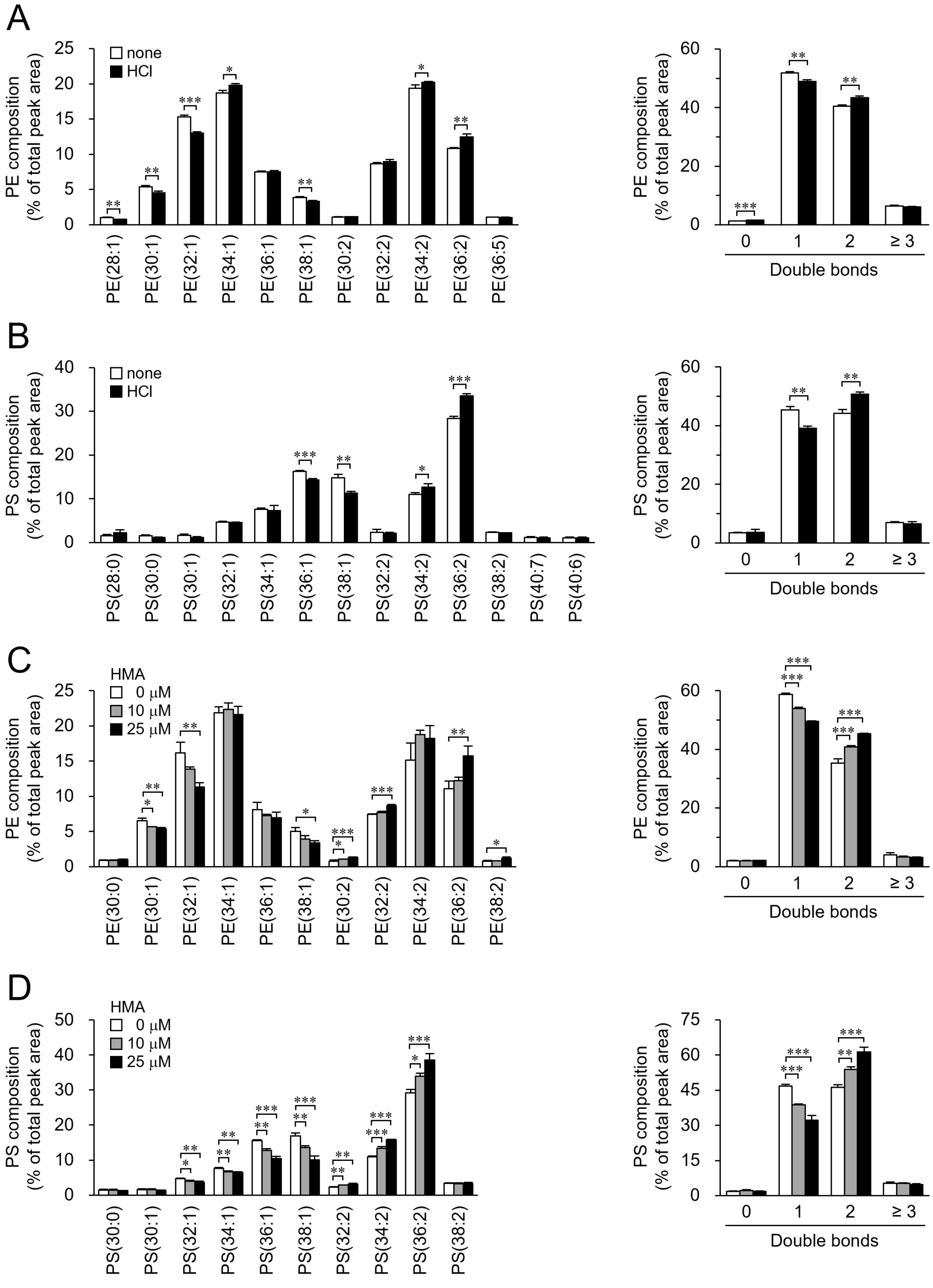

Supplementary Fig. S13. Effect of acidic stress on PE and PS composition.
(*A*,*B*) S2 cells were incubated in culture medium in the presence or absence of 4 mM HCl for 24 h. (*C*,*D*) S2 cells were incubated in culture medium containing indicated concentrations of HMA for 24 h. The molecular composition of PE (*A*,*C*) and PS (*B*,*D*) was analyzed. (*A*-*D*, left) Phospholipid molecules were presented in the format PE(X:Y) and PS(X:Y), where X denotes the total number of acyl chain carbons and Y denotes the total number of double bonds in acyl chains. (*A*-*D*, right) Phospholipid molecules were categorized based on the number of double bonds in their acyl chains. Mean ± SD (n = 3). *$P < 0.05$; **$P < 0.01$; ***$P < 0.001$.

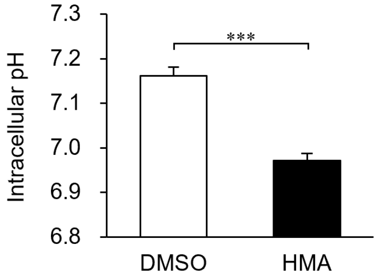

Supplementary Fig. S14. Effect of HMA on intracellular pH.
S2 cells were incubated in culture medium in the presence or absence of 25 μM HMA for 24 h. Intracellular pH were measured. Mean ± SD (*n* = 3). ***$P < 0.001$.

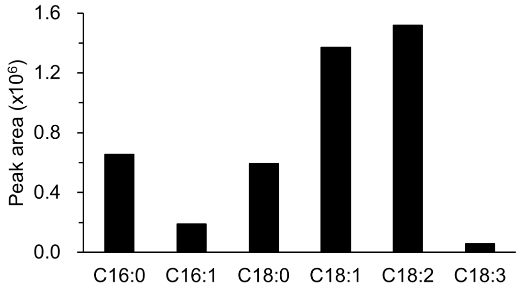

Supplementary Fig. S15. The levels of free fatty acids in fetal bovine serum. The levels of free fatty acids in fetal bovine serum were analyzed.
